# Optimized ND4 allotopic expression for gene therapy of Leber’s hereditary optic neuropathy

**DOI:** 10.3389/fbioe.2026.1765995

**Published:** 2026-03-20

**Authors:** Evgeniy V. Lapshin, Alexander D. Egorov, Alexander S. Malogolovkin, Nizami B. Gasanov, Svetlana A. Smirnikhina, Vyacheslav Yu Tabakov, Alexander V. Karabelsky

**Affiliations:** 1 Department of Gene Therapy, Sirius University of Science and Technology, Sochi, Russia; 2 Sechenov First Moscow State Medical University (Sechenov University), Moscow, Russia; 3 Federal State Budgetary Scientific Institution Research Centre of Medical Genetics, Moscow, Russia

**Keywords:** gene therapy, Leber’s neuropathy, mitochondrial function test, mitochondrial localization, mitochondrial transport

## Abstract

Leber’s hereditary optic neuropathy (LHON) is a mitochondrial disorder characterized by central vision loss, primarily resulting from mutations disrupting the electron transport chain. The most prevalent LHON-causing mutation is mt.11778G>A in the mitochondrial *MT-ND4* gene, which encodes a critical subunit of complex I. Allotopic expression, a promising gene therapy strategy, aims to deliver a functional nuclear version of *ND4* into the cell nucleus and target the resulting protein to the mitochondria. The efficiency of this approach critically depends on the mitochondrial targeting signal used. In this study, we screened five different MTS sequences to optimize the allotopic expression of *ND4* in a HEK-293 cellular model of LHON harboring the mt.11778G>A mutation. We identified MTS-cox8k as the most effective signal for restoring mitochondrial function. Treatment with this construct significantly mitigated key pathological hallmarks: reactive oxygen species decreased by 72%, mitochondrial calcium levels dropped by 47%, and mitochondrial membrane potential (ΔΨm) increased by 38%. These results underscore the therapeutic potential of allotopic *ND4* expression and highlight the critical importance of MTS optimization for developing effective treatments for mitochondrial diseases like LHON.

## 1 Introduction

Leber’s hereditary optic neuropathy (LHON) is a disease that leads to central vision loss. It results from mutations affecting the electron transport chain (ETC), specifically the first complex, which disrupts ATP production, increases the generation of reactive oxygen species (ROS), and causes calcium ions (Ca^2+^) to accumulate in the mitochondria. Retinal ganglion cells (RGCs) are most sensitive to mitochondrial disorders, which lead to their death and eventually to optic nerve atrophy (Yu-Wai-Man et al., 2022).

The most common mutations (approximately 90%) responsible for the manifestation of LHON phenotypes are located in the *MT-ND4* (mt.11778 G>A (70%)), *MT-ND1* (mt.3460 G>A (13%)), and *MT-ND6* (mt.14484 T>C (14%)) genes. The mutation in the *MT-ND4* gene (mt.11778 G>A) is the most common among LHON carriers. This gene encodes NADH dehydrogenase-4, a component of the first complex of the ETC, which provides electron transport. With a prevalence of 1 in 50,000 to 1 in 30,000, LHON manifests at approximately age 15 and most commonly affects men. Central vision loss in LHON occurs first in one eye and only weeks to months later in the other (Yu-Wai-Man et al., 2022). A more severe form of the disease, known as LHON-plus, may also occur. In the presence of additional mutations in the genes of the first ETC complex, not only RGCs are sensitive to LHON-plus, which is characterized by ptosis, dysarthria, dystonia, and recurrent episodes of longitudinally extensive transverse myelitis with residual spasticity (Berardo et al., 2020). Therefore, it is necessary to assess the effect of LHON mutations on cell types other than RGCs but which are similar in some expression characteristics to neuron-like cells. This makes sense, as there is evidence of LHON mutations affecting fibroblasts in the context of reduced oxygen consumption profiles and high growth rates (Uittenbogaard et al., 2019; Nair et al., 2024).

The lack of an approved therapy targeting the pathogenic mechanism of LHON represents a significant unmet medical need. A common treatment is idebenone, an analog of ubiquinone that supports ATP synthesis and has antioxidant properties. Even after taking idebenone, most patients experience irreversible vision loss (Shemesh et al., 2025). In this regard, the development of new methods for treating LHON remains relevant, and one emerging area for this is gene replacement therapy.

Gene therapy using recombinant adeno-associated viral vectors (rAAVs) has shown significant success in treating hereditary retinopathies, laying the foundation for its use in LHON. For example, AAV-mediated therapy successfully restores photoreceptor function in retinal organoids that model Leber’s type 4 amaurosis caused by mutations in the *AIPL1* gene (Sai et al., 2024). In another case, an improved rAAVDJ-3M capsid was used to treat MFRP deficiency-associated retinal degeneration, providing effective and specific transduction of the retinal pigment epithelium upon subretinal injection (Tian et al., 2023). These studies highlight the importance of optimizing both the therapeutic gene and the delivery vector to achieve maximum treatment efficacy.

In the context of these advances, one promising strategy for the use of gene replacement therapy to treat LHON is the use of rAAV to deliver functionally active ND4 to the RGC via intravitreal injection. Since ND4 localizes in the inner mitochondrial membrane, it is necessary to ensure allotopic expression of the transgene—that is, transport of the translated protein into the mitochondria. For this purpose, mitochondrial targeting signals (MTS) are commonly used, as short peptides at the N-terminus of the target protein containing positively charged basic amino acid residues. They are cleaved by mitochondrial processing peptidase after the protein enters the mitochondrial matrix (Bacman et al., 2020). The optimal charge, length, and structure of the MTS depend on the sequence and structure of the transported protein itself (Chin et al., 2018). Therefore, here we present screening data for the most studied MTS (MTS cytochrome oxidase 8 (MTS-cox8k), modified MTS cytochrome oxidase 8: 25 amino acid replacement of lysine K with asparagine N (MTS-cox8n), MTS cytochrome oxidase 10 (MTS-cox10), 4 (MTS-cox4), and MTS of chaperone DNAjc30 (MTS-DNAjc30)) at the N-terminus of ND4 to identify the best candidate for compensating for the effects of LHON. Tests of the functional activity of the MTS-ND4opt transgene included measurement of total cellular ROS, measurement of mitochondrial H_2_O_2_ and Ca^2+^, and mitochondrial membrane potential (ΔΨm).

For broad screening of our genetic constructs, we needed a relevant cell model that could be easily transfected and transduced, with high growth potential, exerting an expression profile similar to neuron-like cells. In general, HEK-293 cells address these requirements, so we generated a new cell line by replacing their mitochondria with mitochondria from LHON patient-derived cells (mt.11778 G>A). This generated cell line is also of interest for research into the effect of the mutation on cell types other than RGCs, as occurs in LHON-plus. In the HEK-293-LHON cell model we generated, which has the mt.11778 G>A mutation, the main molecular features of LHON (according to the tests described) were demonstrated, and the ability of the MTS-ND4opt transgene to compensate for these effects, especially with the participation of MTS-cox8k, was shown. Our data confirm the need to optimize and select MTS for the target peptide. The MTS-cox8k-ND4opt genetic construct we selected to reduce the negative effects of LHON may be an optimal candidate for use in gene therapy for Leber’s neuropathy.

## 2 Results

### 2.1 Genetic construct generation

**FIGURE 1:**
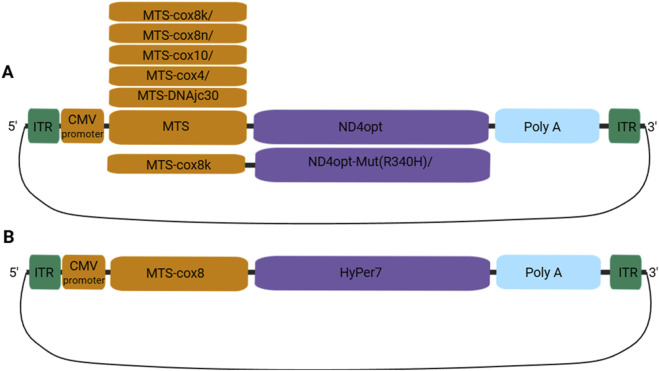
Scheme of the generated expression vectors: **(A)** vectors encoding *ND4opt* with one of the MTS (cox8k/n, cox10, cox4, and DNAjc30); **(B)** vector encoding a genetically encoded hydrogen peroxide sensor (HyPer7) with MTS.

We constructed seven plasmid vectors encoding ND4 fused to distinct N-terminal mitochondrial targeting signal (MTS), including one bearing the R340H mutation. We also generated a plasmid encoding MTS-cox8-HyPer7 (Figure 1).

### 2.2 LHON-specific HEK-293 cell line

**FIGURE 2:**
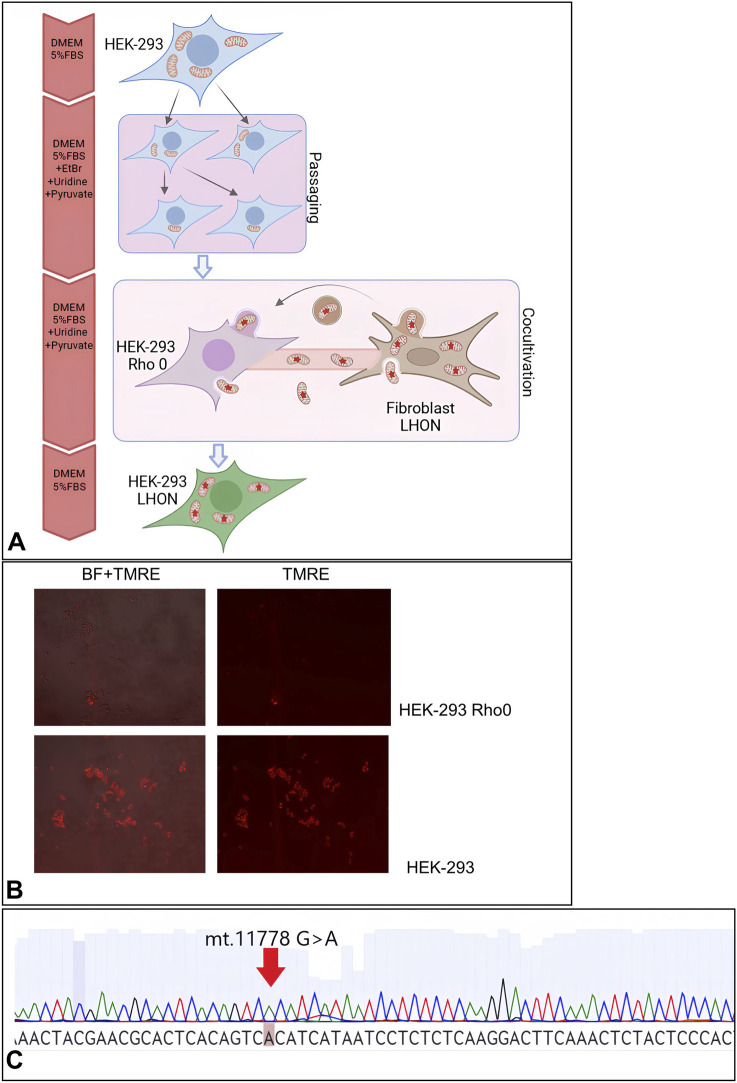
**(A)** Schematic representation of the generation of the HEK-293 LHON cell line with the *MT-ND4* mutation (mt.11778 G>A); **(B)** TMRE fluorescence (accumulates in mitochondria) in native HEK-293 and HEK-293 Rho 0 cells; **(C)** sequence of the *MT-ND4* region.

We depleted mitochondria from HEK-293 cells by incubation with EtBr, uridine, and pyruvate, then introduced mitochondria from LHON (mt.11778 G>A) patient fibroblasts via co-culture. We thus generated a HEK-293 cell line with the *MT-ND4* (mt.11778 G>A) mutation (Figure 2). In the nucleotide sequence of mitochondrial DNA, we observed the substitution of A at position mt.11719 to patient-specific G, which was not detected in parental HEK-293.

**FIGURE 3:**
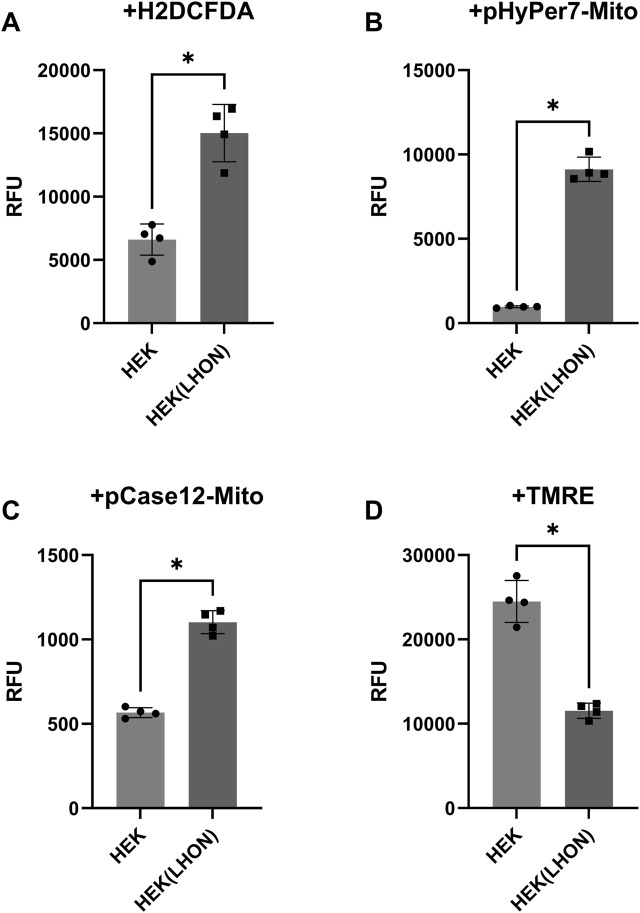
Values of ROS, hydrogen peroxide, calcium ions, and ΔΨm levels in HEK-293 (HEK) and HEK-293 LHON (HEK(LHON)) cell lines. **(A)** DCF (ROS) fluorescence level in cells; **(B)** HyPer7 fluorescence level (hydrogen peroxide) in mitochondria; **(C)** Case12 fluorescence level (calcium ions) in mitochondria; **(D)** TMRE fluorescence level (ΔΨm) in cells; n = 4 biological replicates. Data are shown as means ± SD. In each panel, statistics were evaluated using the Mann–Whitney U test; *p < 0.05.

This newly developed HEK-293-LHON cell line demonstrated increased levels of total cellular ROS (by 128%), mitochondrial hydrogen peroxide (by 838%), and mitochondrial calcium ions (by 95%) and decreased ΔΨm (an indicator of reduced ATP generation) (by 53%) compared to the original HEK-293 cells (Figure 3).

### 2.3 *ND4opt* gene delivery regulates the level of ROS, calcium ions, and ΔΨm in HEK-293-LHON cells

#### 2.3.1 Total ROS level

**FIGURE 4:**
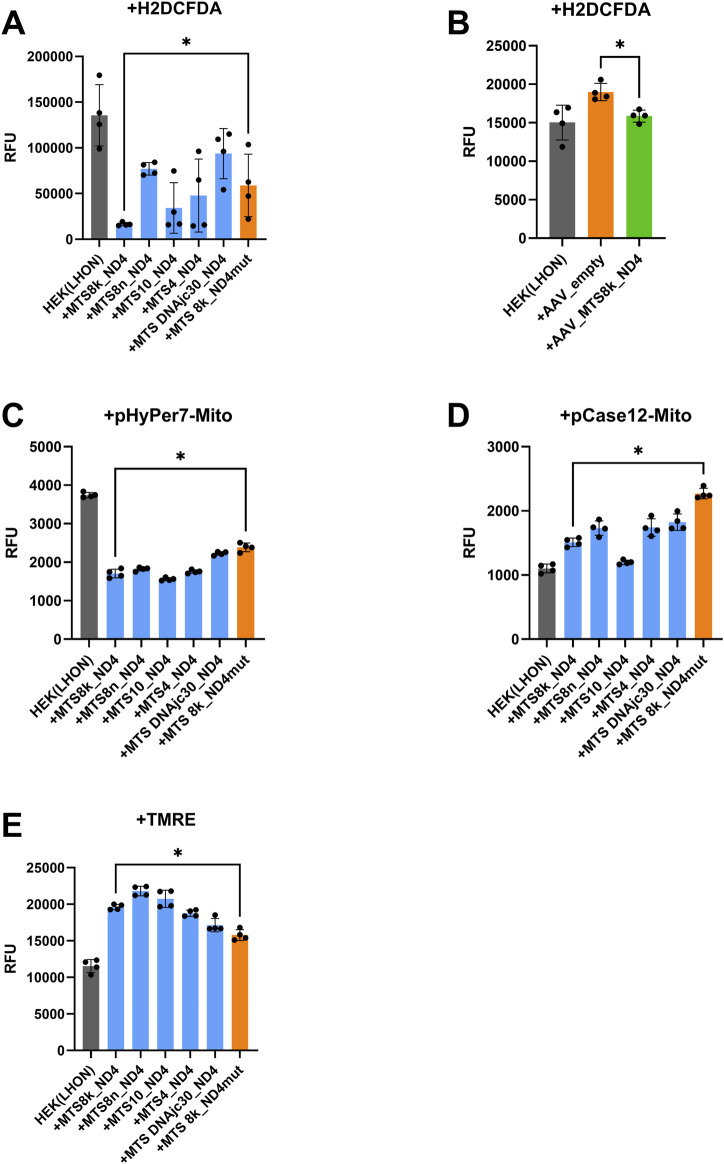
Effects of delivery of the functionally active *ND4opt* gene with various MTS into HEK-293 (LHON) cells on the level of ROS, hydrogen peroxide, calcium ions, and ΔΨm; **(A)** DCF (ROS) fluorescence levels in cells after delivery of the *ND4opt* gene with different MTS using plasmid vectors; **(B)** DCF fluorescence level (ROS) in cells after delivery of the *ND4opt* gene with various MTS using AAV vectors; **(C)** HyPer7 fluorescence level (hydrogen peroxide) in mitochondria after delivery of the *ND4opt* gene with various MTS using plasmid vectors; **(D)** Case12 (calcium ion) fluorescence level in mitochondria after delivery of the *ND4opt* gene with various MTS using plasmid vectors; **(E)** TMRE (ΔΨm) fluorescence level in cells after delivery of the *ND4opt* gene with different MTS using plasmid vectors; n = 4 biological replicates. Data are shown as means ± SD. In each panel, statistics were evaluated using the Mann–Whitney U test, where each variant “+MTSx_ND4” was compared with “+MTS8k_ND4mut” or AAV_empty (for **B**), and the effect of the variant on the figure (shown by *) is indicated only for the variant “+MTS8k_ND4”; *p < 0.05.

The total level of ROS in HEK-293-LHON cells, expressed as DCF (deacetylated, oxidized form of H2DCFDA) fluorescence intensity, decreased by 72% in response to transfection with a plasmid vector encoding ND4 with MTS cytochrome oxidase 8 (MTS8k-ND4opt) at the N-terminus of the amino acid chain compared to transfection with a control plasmid encoding a defective version of ND4 (R340H) (P value < 0.05) (Figure 4A). A similar effect of reducing the level of ROS is observed in the case of the transduction of AAV carrying the MTS8k-ND4opt genetic construct by 16% compared to the control variant with empty AAV capsids (P value < 0.05) (Figure 4B). In the case of *ND4opt* with MTS cytochrome oxidases 10 and 4, a statistically insignificant decrease in ROS was observed (P value >0.05), while in the case of MTS cytochrome oxidase 8n and DNAjc30 chaperone, the ROS level increased.

#### 2.3.2 Mitochondrial hydrogen peroxide level

The level of hydrogen peroxide in the HEK-293-LHON cells, determined by the fluorescence intensity of the HyPer7 sensor (directed to mitochondria using MTS cytochrome oxidase 8), decreased by 53% after delivery of the *ND4opt* gene with MTS cytochrome oxidase 8k, 8n, 10, 4, and DNAjc30 chaperone using a plasmid vector, compared to the control variant in which the functionally active gene was replaced with a defective *ND4opt* with the R340H mutation (Figure 4C).

#### 2.3.3 Calcium ion levels in HEK-293 (LHON) mitochondria

The level of calcium ions in the HEK-293-LHON cells, determined by the fluorescence intensity of the Case12 sensor (directed to mitochondria using MTS cytochrome oxidase 8), decreased by 47% after delivery of the *ND4opt* gene with MTS cytochrome oxidase 8k, 8n, 10, 4, and DNAjc30 chaperone protein using a plasmid vector, compared to the control variant in which the functionally active gene was replaced with a defective *ND4opt* with the R340H mutation (P value < 0.05) (Figure 4D).

#### 2.3.4 ΔΨm level

The ΔΨm level (an important characteristic created by the ETC as a result of pumping protons into the intermembrane space, creating the proton gradient necessary for ATP synthase to produce ATP), determined by the TMRE fluorescence intensity level, increased by 38% after delivery of the *ND4opt* gene with MTS cytochrome oxidase 8k, 8n, 10, and 4 using a plasmid vector, compared to the control variant in which the functionally active gene was replaced with a defective *ND4opt* with the R340H mutation (P value < 0.05) (Figure 4E).

## 3 Discussion

In this study, we created a HEK-293 cell-based model carrying mitochondria from LHON patients with the mt.11778G>A mutation in the *MT-ND4* gene (HEK-293-LHON).

HEK-293 cells are widely used in gene therapy research for their high transfection and transduction efficiency. Moreover, they endogenously express several neuronal proteins and may have partial neuronal lineage features, supporting their use for basic neurobiology and channel/receptor pharmacology assays. This neuron-like expression profile makes them a valuable tool for neuropathy research (He and Soderlund, 2010).

Our cell model reproduced key biochemical and functional abnormalities characteristic of neuronal cell damage in LHON, including increased levels of ROS, impaired calcium homeostasis, and decreased ΔΨm.

The data generated in this cell model confirm that mitochondria with the *MT-ND4* mutation cause oxidative stress, which is consistent with previous studies demonstrating the role of complex I defects in ROS generation (Stenton et al., 2021). Elevated levels of ROS in cells, measured using H2DCFDA, and mitochondrial hydrogen peroxide, detected by the HyPer7 sensor, indicate ETC dysfunction. This supports the hypothesis that the mt.11778 G>A mutation disrupts normal electron flow in mitochondrial ETC, leading to increased ROS levels and mitochondrial DNA damage (Peng and Mei-Jie, 2004).

In addition, we showed that this mutation increases the level of calcium ions (Ca^2+^) in mitochondria, as measured by Case12 sensor. This effect could be related to the destabilization of mitochondrial calcium buffering due to reduced ΔΨm and the opening of the mitochondrial transition pore (mPTP), which may exacerbate cellular stress (Sedlic et al., 2010).

Measurements using TMRE in HEK-293-LHON cells showed a decrease of ΔΨm in cells with the mutant *MT-ND4* variant. This is consistent with the known mechanism of LHON, in which mutations in *MT-ND4* are proposed to disrupt proton transport from complex I, reducing the efficiency of oxidative phosphorylation (Crowley et al., 2016).

Although the proposed *in vitro* model successfully reproduces key aspects of LHON, it should be noted that it does not fully mimic the retinal neurons that are most vulnerable in LHON. For a deeper understanding of the pathogenesis of LHON and efficiency of gene therapy, it is necessary to create neuronal models (e.g., differentiated iPSCs into retinal ganglion cells).

We demonstrated that allotopic expression of *ND4opt* using various MTS (cytochrome oxidases 8k, 8n, 10, 4, and chaperone DNAjc30) compensates for key biochemical defects caused by the *MT-ND4* mt.11778 G>A mutation in the HEK-293 (LHON) cell model. Our results show a significant improvement in key parameters of mitochondrial function in this model, supporting the potential of using these MTS for the further development of LHON gene therapy approaches.

The observed decrease in DCF fluorescence (deacetylated, oxidized form of H2DCFDA) indicates normalization of the level of active oxygen species in the cytosol in our HEK-293 (LHON) model. This effect is particularly pronounced when using MTS cytochrome oxidase 8, which directs ND4 to the mitochondrial matrix (MTS8k-ND4opt) in both transfection and rAAV transduction. It is important to note that a similar decrease in fluorescence was also recorded for the mitochondria-targeted HyPer7 sensor, indicating the restoration of redox status directly in the mitochondrial matrix of these cells.

The decrease in Case12 signal in mitochondria after the introduction of the therapeutic construct indicates the restoration of the ability of mitochondria to maintain optimal calcium ion levels in this cellular context. This effect may be associated with improved function of complex I of the respiratory chain, leading to the restoration of ΔΨm and, as a result, normalization of mitochondrial calcium transporters. These findings are particularly important given the key role of calcium homeostasis disruption in the pathogenesis of LHON, although confirmation in neuronal cells is required.

The increase in TMRE fluorescence clearly indicates the restoration of ΔΨm in all experimental groups. This suggests an enhancement of proton transport from complex I of the ETC due to allotopic expression of functional ND4 in our experimental system.

In this study, we evaluated alternative MTSs in a series of analyses that simulated different stages of the pathological cascade in LHON using a HEK-293-based model. To come to a unified conclusion, we established a hierarchy of criteria based on the known pathogenesis of the disease. The primary factor was recognized as a decrease in ROS levels, since oxidative stress is the initiating event. The MTS-cox8k variant consistently demonstrated superiority in this key test. Although in other analyses, some MTS, especially MTS-cox10, showed comparable or even slightly better efficacy, their suboptimal activity against ROS calls into question their ability to address the root of the problem. The secondary improvements in ΔΨm and Ca^2+^ under the action of MTS-cox8k-ND4opt logically follow from its ability to neutralize the primary stress in this model. Thus, a comprehensive analysis within the scope of our cellular model points to MTS-cox8k as a promising candidate for further development.

Our results demonstrate that the MTS sequence significantly affects therapeutic efficacy in the HEK-293-LHON model. This is consistent with current understanding of the importance of proper targeting of allotypically expressed proteins for their functional activity (Chin et al., 2018). This effect may be due to more efficient targeting of ND4 to the mitochondrial matrix, where the initial assembly of complex I occurs, an optimal balance between the rate of import and subsequent protein assembly, an optimal ratio of hydrophobicity and charge of the sequence, and a lower tendency to form aggregates during import. MitoFates analysis (Fukasawa et al., 2015) shows that MTS-cox8k and MTS-cox10 sequences having extended regions with a high “Max positively charged amphiphilicity” score are characterized by two adjacent cleavage sites for the peptidases MPP and Icp55 and have a low net charge (0.091 for MTS-cox8, 0.083 for MTS-cox10, 0.214 for MTS-cox4, and 0.227 for MTS-DNAjc30). Taken together, these characteristics might contribute to more optimal transport into mitochondria (Supplementary 4).

Intriguingly, our results revealed that the introduction of the mutant ND4 variant (R340H) into the HEK-293 (LHON) cells model partially restored mitochondrial function. This restoration was evidenced by decreased ROS levels and increased ΔΨm, although elevated intramitochondrial calcium concentrations were also elevated. These findings suggest that the ND4 R340H protein variant may retain partial activity. Presumably, the defective ND4 (R340H) protein maintains a limited ability to integrate into Complex I, thereby contributing to proton translocation and electron transport, albeit with reduced efficiency.

## 4 Materials and methods

### 4.1 Genetic constructs

#### 4.1.1 ND4opt

The annotation of the *MT-ND4* gene coding sequence was performed using the NCBI/CDD service. The UniPro UGENE tool was used for nucleotide sequence alignment (Okonechnikov et al., 2012). The nucleotide sequence was translated into the amino acid sequence using the EMBOSS Transeq tool (EMBL-EBI and Institute, n.d.).

To study transcript stability, the RNAfold program was used (RNAfold Web Server, n.d.). The nucleic acid with the optimized *MT-ND4* gene sequence (*ND4opt*, Supplementary 1), encoding the ND4 protein, was custom-synthesized using a service from Top Gene Technologies (Canada). Additionally, analysis of the protein’s unstructured regions was performed to select optimal regions for optimization using IUPred2A (Dosztányi, 2018).

#### 4.1.2 Creation of *MTS-ND4opt* constructs

The MTS was identified, and the mutational landscape and the MTS processing efficiency were analyzed in MitaViewer (MTSviewer, n.d.) and МitoFates (Fukasawa et al., 2015). Synthesized MTS with a complementary region 3′ to 5′ ND4opt were used as primers for amplification of the MTS-ND4opt sequence.

Plasmid expression vectors pAAV-MTS-ND4opt, expressing *ND4opt* with one of the variants of the MTS-encoding sequence, were generated by inserting the MTS-ND4opt sequence into the pAAV plasmid vector for virus assembly. DNA with the MTS-ND4opt sequence was generated by PCR with sequence overlap (splicing by overlap extension/(SOE) PCR).

DNA with the MTS sequence (MTS-cox8k, MTS-cox8n, MTS-cox4, MTS-cox10, and MTS-DNAjc30; Supplementary Table S1) was combined with DNA with the *ND4opt* sequence generated, as described above using the primers listed in Supplementary Table S1. The primers contained a short MTS sequence at the 5' to 3' end and a forward primer sequence for *ND4opt*.

In the first stage, one of the forward primers was used: MTS-cox8n, MTS-cox8k, MTS-cox10, MTS-cox4, and MTS-DNAjc30 for the corresponding MTS fragment and the reverse primer ND4_opt_rev, with *ND4opt* used as the matrix. Amplification program: preliminary DNA melting at 98 °C for 30 s, 15 amplification cycles including melting for 10 s at 98 °C, annealing for 30 s at 55 °C, and elongation at 72 °C for 30 s; final elongation at 72 °C for 3 min.

In the second stage, one of the forward primers was used: MTS-COX8-BamHI_Fw, MTS-COX-4-OPT-Fw, MTS-COX-10-OPT-Fw, and MTS-DNAjc30-Fw for the corresponding MTS fragment and the reverse primer ND4_opt_rev, using the PCR product from stage 1 as a template. Amplification program: preliminary DNA melting at 98 °C for 30 s, 30 amplification cycles including melting for 10 s at 98 °C, annealing for 30 s at 55 °C, and elongation at 72 °C for 30 s; final elongation at 72 °C for 3 min.

#### 4.1.3 Assembly of the expression vector pAAV-MTS-ND4opt

The amplified fragments MTS-cox4-ND4, MTS-cox10-ND4, MTS-cox8k-ND4, MTS-cox8n-ND4, and MTS-DNAjc30-ND4 were cloned into the pAAV-CMV-MCS vector (Cell Biolabs, cat. VPK-410). Restriction digestion was performed according to the method recommended by the manufacturer of pAAV-CMV-MCS using BamHI and HindIII restriction endonucleases (NEB, cat. R0136S and R0104S). The ligation reaction of the prepared inserts and vectors was performed according to the manufacturer’s recommended protocol in 20 μL. For ligation, 10× T4 DNA Ligation Buffer and T4 DNA Ligase (NEB, cat. M0202S) were used, and the reaction was carried out at 16 °C overnight. The enzyme was inactivated at 65 °C for 10 min.

Cell transformation was performed according to the standard protocol (Molecular Cloning, n.d.). To perform the transformation, an aliquot of chemically competent *Escherichia coli* DH5α cells (NEB, cat. C2987H) was removed from the freezer (−80 °C) and placed on ice for slow thawing. We added 5 μL of cooled ligase mixture to the thawed cells and incubated this on ice for 30 min. Heat shock was performed for 30 s at 42 °C in a water bath, then the sample tube with cells was transferred to ice and incubated for 2 min. Thence, 1 mL of SOC media (NEB, cat. B9020S) preheated to 37 °C was added to the cells and incubated in a thermostat for 1 h at 37 °C with stirring at 180–200 rpm. The suspension of transformed cells was seeded onto Petri dishes pre-dried in a thermostat at 37 °C with LB agar (neoFroxx, cat. 1311KG2P5) and ampicillin (CDH, cat. TC1021) at 100 μg/mL. The cells were cultured in a thermostat at 37 °C for 16–18 h.

To analyze the colonies of transformed cells, PCR screening was performed using vector-specific primers pAAV_For_seq2 and pAAV_Rev_seq2 (Supplementary Table S2) and a ready-to-use PCR mixture ScreenMix (Evrogen, cat. PK041). The reaction mixture was prepared according to the manufacturer’s recommendations. Thermolysates of individual bacterial colonies were used as the matrix. To do this, cells from each colony were collected from the surface of the culture medium using a micropipette tip and transferred to a test tube containing 20 μL of water. The test tubes containing the bacterial suspension were heated at 95 °C for 5 min. In PCR, 2 μL of suspension was taken. PCR was performed with the following parameters: preliminary melting of DNA at 95 °C for 3 min, 25 amplification cycles, including melting for 20 s at 95 °C, annealing for 20 s at 55 °C, and elongation at 72 °C for 2 min; final elongation at 72 °C for 5 min. Clones for which the presence of an insert of the correct size was confirmed by PCR were sequenced. Clones with the correct sequence were selected based on the sequencing results.

#### 4.1.4 Generating a nucleotide substitution in ND4opt similar to mt.11778 G>A

A nucleotide substitution was introduced into the previously generated expression plasmid MTS-cox8k-ND4opt by PCR using specific primers, which were used as a template for site-directed mutagenesis. The GG>AC substitution was introduced at the position of two nucleotides *ND4opt* (340 amino acid (R340H)). This substitution corresponds to a mutation in the *MT-ND4* gene in LHON, where arginine is replaced by histidine, leading to the dysfunction of NADH dehydrogenase-4.

The primers for substitution were selected in such a way as to amplify halves of the original plasmid, the ends of which would be complementary to each other over the length of the selected primers (Supplementary Table S3).

PCR was performed using Tersus polymerase (Evrogen, cat. PK221) with the following parameters: preliminary DNA melting at 95 °C for 2 min, 30 amplification cycles, including melting for 15 s at 95 °C, annealing for 15 s at 63 °C, and elongation at 72 °C for 3 min; final elongation at 72 °C for 2 min.

The amplified DNA fragments were separated in a 1% agarose gel at an electric field voltage of 80 V, and then the isolated fragments were purified using a CleanUp kit (Evrogen, cat. BC022). The purified fragments were then used for transformation into the *E. coli* DH5α strain. The resulting plasmids were verified by Sanger sequencing.

#### 4.1.5 Cloning of MTS8-HyPer7

The HyPer7 sequence was amplified from the source plasmid pCS2+HyPer7 (Addgene, cat. 136466) using primers (Supplementary Table S4) with HindIII restriction sites at 3′ and Bgl II at 5′. MTS8 (from cytochrome oxidase 8) was amplified from the source plasmid pHyPer-dMito (Evrogen, cat. FP942) using primers (Supplementary Table S4) with Sal I restriction sites at 3′ and HindIII at 5′. Sequences were amplified using Tersus polymerase (Evrogen, cat. PK221) according to the manufacturer’s protocol, PCR mode: preliminary DNA melting at 95 °C for 2 min, 35 amplification cycles, including melting for 10 s at 95 °C, annealing for 10 s at 57 °C (for MTS-cox8) or 55 °C (for HyPer7), and elongation at 72 °C for 20 s (for MTS-cox8) or 1 min 30 s (for HyPer7); final elongation at 72 °C for 1 min. The fragments were purified using the CleanUP kit (Evrogen, cat. BC022) and the fragments and vector for insertion pAAV-MCS Expression Vector (Cell Biolabs, cat. VPK-410) were restricted with Sal I, HindIII, and Bgl II (NEB, cat. R0138, R0104, and R0144, correspondingly) according to the manufacturer’s protocol. The fragments were purified using the CleanUP kit (Evrogen, cat. BC022), and the ligation reaction was performed in a molar ratio of MTS-cox8, HyPer7, and pAAV-MCS Expression Vector fragments in a 5:5:1 ratio, respectively, with T4 ligase (NEB, cat. M0202S) according to the manufacturer’s protocol. Competent cells were transformed as described earlier.

### 4.2 HEK-293-LHON mt.11778 G>A cell line

The HEK-293 cell line (ATCC cat. CRL-1573) was transferred to DMEM medium containing 4.5 g/L glucose (Biosera cat. PM-D1115), 5% v/v FBS (Biowest, cat. S1600), 2.5 mM pyruvate (PanEco, cat. F023), 1× NEAA (PanEco, cat. F115), 100 μg/mL uridine (Sigma-Aldrich, cat. U3750), and 25 ng/mL EtBr (Sigma-Aldrich, cat. 46067) with a monthly increase in concentration to 50, 100, 200, 300, and finally 400 ng/mL (King and Attardi, 1989). Mitochondrial removal was verified using the voltage-dependent mitochondrial dye TMRE. Thence, the HEK-293 Rho 0 cell line was cultured without the addition of EtBr.

By co-cultivation of HEK-293 Rho 0 cells with LHON fibroblast cell culture (generated from a patient with Leber’s neuropathy, with *MT-ND4* mt.11778 G>A mutation), mitochondrial transfer was induced (Zhang and Miao, 2023). The study was approved by the Ethics Committee of the Research Centre for Medical Genetics (Moscow, Russia) and conducted in accordance with provisions of the Declaration of Helsinki of 1975. The patient signed an informed written consent form as an anonymous participant of the study and a donor of biological materials. LHON fibroblast culture was obtained from biopsy material using Amniocar proliferative medium (PanEco, Russia). Primary fibroblast cells were obtained and deposited in the Moscow Branch of the Biobank “All-Russian Collection of Biological Samples of Hereditary Diseases”. One million HEK-293 Rho 0 cells were seeded onto a 25-cm^2^ culture flask, in which LHON fibroblasts were cultured at 80% confluence in a medium without EtBr. Since HEK-293 Rho 0 cells and LHON fibroblasts differ greatly in morphology and trypsinization time, the cells were separated during passaging based on these characteristics. Every 3 days, 1/10 of the HEK-293 Rho 0 cells were passaged back into the flask for 30 days. LHON fibroblasts were replaced with fresh ones three times during co-cultivation. Mitochondrial transfer was determined by TMRE staining. The presence of the mt.11778 G>A mutation in the mitochondrial genome of individual HEK-293 LHON clones was determined by Sanger sequencing of the amplified *MT-ND4* region.

### 4.3 Viral vectors

The suspension HEK-239 cells were transfected with three plasmids: pAAV with the gene of interest surrounded by ITR, pHelper (Cell Biolabs, cat. 340202) containing the E4, E2a, and VA genes, pRepCap 2/9 (Addgene, cat. 112865) encoding the Rep 2 serotype life cycle genes and the Cap 9 serotype capsid genes. The molar ratio of plasmids was 2:2:5, correspondingly. The DNA was mixed with PEI MAX (Polysciences, cat. 24765) in a mass ratio of 1:5, correspondingly, to a final volume of 5% (DNA and PEI MAX were diluted in Opti-MEM (Gibco, cat. 31985062)) of the HEK-293 culture medium volume. The amount of DNA was determined based on the number of HEK-293 cells, at a rate of 1.5 μg per 1 million cells.

After transfection, the HEK-293 culture was incubated at 37 °C, 5% CO_2_, with orbital shaking at 100 rpm for 120 h. After incubation, the cells were lysed by adding Tween-20 (Sigma-Aldrich, cat. P1379) to 0.05% for 1 h at 37 °C and at 100 rpm. Then, MgCl_2_ (Sigma-Aldrich, cat. 8.14733) was added to 1 mM and Benzonase (Dia M, cat. 3549.0025) to 30 EA per mL, incubated for 1 h at 37 °C and at 100 rpm. The lysate was centrifuged at 3,000 g for 10 min, the supernatant was passed through a depth filtration system with kieselguhr (Sigma-Aldrich, cat. 1.07910) 1.5 g per 100 mL of liquid and through a 0.22 μm membrane (TPP, cat. 99150). The filtrate was concentrated in a tangential filtration system (Sartorius, cat. VF20P4) with a separation limit of 100 kDa. The concentrate was chromatographically purified on AAVX sorbent (Thermo Fisher Scientific, cat. A36740). The number of viral genomes in the sample was determined by quantitative PCR.

### 4.4 Transduction

A viral preparation was added to the cell suspension at a rate of 100,000 viral genomes per cell. The resulting suspension (150,000 HEK-293-LHON cells per well) in DMEM (Biosera cat. PM-D1115), 2% FBS (Biowest, cat. S1600), was transferred to a 24-well plate and incubated at 37 °C, 5% CO_2_. The next day, the growth medium was replaced with complete medium.

### 4.5 Transfection

In a 24-well plate, 150,000 HEK-29-LHON cells were seeded by suspension into each well immediately before transfection, in 500 µL medium; 150 ng of DNA was used for transfection. The DNA dissolved in 25 µL of DMEM per well was mixed with 0.45 µL of FuGENE HD (Promega, cat. HD-1000). The mixture was incubated at room temperature for 12 min and added to the cell suspension. After pipetting, the cells were transferred to the wells of the culture plate. The next day, the growth medium was replaced with complete medium.

### 4.6 Functional test with genetically encoded sensors

The plasmid encoding the ROS sensor (MTS-cox8-HyPer7) or calcium ions (Case12-mito (Evrogen, cat. FP992)) in a 120 ng/well was co-transfected with the *ND4opt* plasmids a 180 ng/well, as described above.

On the third day after transfection, the cells were analyzed by flow cytometry on a CytoFLEX B5-R3-V5 (Beckman Coulter) based on the sensor’s fluorescence signal.

### 4.7 H2DCFDA functional test

On the third day after transfection with *ND4opt* plasmids, the cells were washed twice with PBS, and 500 μL of a 5 μM H2DCFDA solution in PBS was added. The cells were incubated for 30 min at 37 °C, 5% CO_2_, in the dark, and fluorescence was measured by flow cytometry on a CytoFLEX B5-R3-V5 (Beckman Coulter) based on the DCF fluorescence signal.

### 4.8 TMRE functional test

On the third day after transfection with *ND4opt* plasmids, the cells were washed twice with serum-free DMEM, and 500 μL of a 200 nM TMRE solution in serum-free DMEM was added. The cells were incubated for 20 min at 37 °C, 5% CO_2_, in the dark, and fluorescence was measured by flow cytometry on a CytoFLEX B5-R3-V5 (Beckman Coulter) based on the fluorescence signal of the sensor.

### 4.9 Statistical processing

Statistical analysis was performed using GraphPad Prism 9 software. Statistical significance was set at p < 0.05 based on Mann–Whitney U test calculations. Errors reflect standard deviation. All experimental points in the sample represent biological replicates of the experiments (independent cell transfection/transduction procedure, with parallel execution of the entire measurement cycle for all compared groups).

## Data Availability

The original contributions presented in the study are included in the article/Supplementary Material; further inquiries can be directed to the corresponding author.
