# Supplementary material for: Optimized ND4 allotopic expression for gene therapy of Leber’s hereditary optic neuropathy
Source: Front Bioeng Biotechnol. 2026 Mar 20;14:1765995. doi: 10.3389/fbioe.2026.1765995 (PMC13047110; doi:10.3389/fbioe.2026.1765995)

Supplementary Material

1 Optimized ND4 gene sequence (*ND4opt*)

ctgaaactga tcgtgccaac aattatgctg ctgccactga cctggctgag caagaagcac 60

atgatctgga tcaacaccac cacccacagc ctgatcatca gcatcatccc cctgctcttt 120

ttcaaccaga tcaacaacaa cctgttcagc tgcagcccta ccttctccag cgaccccctg 180

accacacctc tgctgatgct gacaacatgg ctgctgcctc tgaccatcat ggccagccaa 240

agacacctgt caagcgagcc tctgagcaga aagaagctgt atctgagcat gctgatctcc 300

ctgcaaatca gcctcatcat gacctttacc gccaccgagc tgatcatgtt ctacatcttc 360

ttcgagacaa ccctcattcc taccctggcc atcatcacca gatggggcaa ccagcctgag 420

aggctgaacg ccggtacata cttcctgttt tacacacttg tgggcagcct gcctctgcta 480

atcgccctga tctacaccca taataccctg ggatctctta acatcctgct gctgacgctt 540

acagcccagg agctgagcaa cagctgggcc aacaacctga tgtggctggc ctacaccatg 600

gctttcatgg tcaagatgcc tctgtacggc ctgcacctgt ggctgcccaa ggcccacgtg 660

gaagccccta tcgccggcag catggtgctg gccgccgtgc tgctcaagct gggcggctac 720

ggcatgatgc ggctgaccct gatcctgaat cctctgacta agcacatggc ctaccccttc 780

ctggtgctga gcctgtgggg aatgatcatg acatctagca tctgtctgag acagactgat 840

ctgaagagcc tgatcgccta ttcttctatc agccacatgg ctctggtggt gaccgctatc 900

ctgattcaga ccccttggtc ctttaccggc gctgtgatcc tgatgatcgc acacggcctg 960

accagcagcc tgctgttctg cctggctaat tctaattacg agagaacaca tagccggatc 1020

atgatcctgt cccagggcct gcagaccctt ctgcctctga tggccttctg gtggctgctg 1080

gcctctctcg ccaacctggc tctgccacct accattaacc tgctgggcga actgtccgtg 1140

ttagttacca cattcagctg gagcaacatc accctgttac tgaccggcct gaatatgctg 1200

gtgaccgccc tctacagcct gtacatgttc accaccaccc agtggggctc tctgacgcac 1260

cacatcaaca acatgaaacc cagcttcaca cgggaaaaca cactgatgtt catgcacctg 1320

tctcccatcc tgctgctgtc cctgaacccc gacatcatca caggatttag ctcctaatga 1380

2 Primer sequence

Table S1 - Sequence of MTS and primers for their amplification

Title	Sequence
MTS-COX8-BamHI_Fw	ATATATGGATCCATGTCCGTCCTGA
MTS-cox8n	ATATATGGATCCATGTCCGTCCTGACG CCGCTGCTGCTGCGGGGCTTGACAGGCTCGGCCC GGCGGCTCCCAGTGCCGCGCCAACCTGAAAC TGATCGTGCCAAC
MTS-cox4	ATATATGGATCCATGTTGGCTACCAGG GTATTTAGCCTAGTTGGCAAGCGAGCA ATTTCCACCTCTGTGTGTGTACGACTGAAACTGAT CGTGCCAAC
MTS-cox8k	ATATATGGATCCATGTCCGTCCTGACG CCGCTGCTGCTGCGGGGCTTGACAGGCTCGGCCC GGCGGCTCCCAGTGCCGCGCCAAGCTGAAAC TGATCGTGCCAAC
MTS-cox10	ATATATGGATCCATGGCCGCATCTCCG CACACTCTCTCCTCACGCCTCCTGACAGGTTGCG TAGGAGGCTCTGTCTGGTATCTTGAAAGAAGAAC TCTGAAACTGATT GCCAAC
MTS-DNAJC30	ATATATggatccATGGCCGCCATGC GCTGGCGATGGTGGCAGCGGCTG TTACCTTGGAGGTTGCTGCAGGCC CGTGGCTTTCCACAAAATTCTGCA CCCAGCCTGGGCCTGGGCGCCCGC ACCTACCTGAAACTGATCGTGCCAAC
MTS-DNAJC30-Fw	ATATATggatccATGGCCGCCATGCGCTGGCG
MTS-COX-4-OPT-Fw	ATATATGGATCCATGTTGGCTACCAG
MTS-COX-10-OPT-Fw	ATATATGGATCCATGGCCGCATC
ND4_opt_rev	ATATATAAGCTTTCATTAGGAGCTAAA TCCTGTGATG

Table S2 - Primers for sequencing the pAAV-CMV-MCS sequence

Title	Sequence
pAAV_For_seq2	tcttcctcccacagctcctg
pAAV_Rev_seq2	ttccagggccaggagaggca

Table S3 - Primers for substitution in ND4opt as part of the expression plasmid for AAV assembly

Title	Sequence
F (R340H)	GTCTCGCGGTATCATTGCAGCACTG

R1 (pAAV)	CTGGGACAGGATCATGATGTGGCTATGTGTTCTCTCGTAATTAG
F2 (pAAV)	CTAATTACGAGAGAACACATAGCCACATCATGATCCTGTCCCAG
R2 (R340H)	CAGTGCTGCAATGATACCGCGAGAC

Table S4 - Primer sequence for cloning MTScox8-Hyper7

Title	Sequence
Forward MTScox8 (Sal I)	atatatGTCGACATGTCCGTCCTGACGCC
Reverse MTScox8 (HindIII)	atatatAAGCTTATCCCCCAACGAATGGATCTTGG
Forward HyPer7 (HindIII)	atatatAAGCTTatgcacctggctaatgaggag
Reverse HyPer7 (Bgl II)	atatatAGATCTtcaatcgcagatgaagctaacac

3 Flow cytometry data

Table S5 - Data for Figure 3 A

HEK	HEK(LHON)
7771,5	16357,8
6721,8	16937,5
4871	11873
7045,2	14920,4

Table S6 - Data for Figure 3 B

HEK	HEK(LHON)
897,4	8924,8
979,8	8841,1
972,6	8557,3
1042,4	10169,2

Table S7 - Data for Figure 3 C

HEK	HEK(LHON)
573,7	1070,8
530,7	1021,6
601,3	1169,9
560,8	1147,2

Table S8 - Data for Figure 3 D

HEK	HEK(LHON)
21435,4	11401,6
24636,7	12086,5
24390,1	10343,3
27533,3	12365,7

Table S9 - Data for Figure 4 A

HEK(LHON)	+MTS8k_ND4	+MTS8n_ND4	+MTS10_ND4	+MTS4_ND4	+MTS DNAjc30_ND4	+MTS 8k_ND4mut
125679,1	15737,4	70292	16679,1	14536,7	109336,9	22135,7
138273,8	19183,6	81403	74732,5	96232,9	115019,9	103660,1
98922,4	16294,5	84209,3	29760,4	15717	96147,4	62561,8
179412	15096,3	72438,4	15867,9	64987,9	54321,9	47311,5

Table S10 - Data for Figure 4 B

HEK(LHON)	+AAV_empty	+AAV_MTS8k_ND4
16357,8	18875,6	16733,5
16937,5	18399,1	15947,5
11873	18054,5	15928
14920,4	20590,6	14835,8

Table S11 - Data for Figure 4 C

HEK(LHON)	+MTS8k_ND4	+MTS8n_ND4	+MTS10_ND4	+MTS4_ND4	+MTS DNAjc30_ND4	+MTS 8k_ND4mut
3683,7	1839,7	1866,3	1521,1	1707,9	2261	2507,7
3708,8	1747,1	1837,9	1610,2	1758,6	2270,4	2238

3747,5	1585,7	1823,9	1535,4	1816,6	2170,1	2381,1
3835,3	1649	1753,6	1566,2	1741,3	2210	2413,7

Table S12 - Data for Figure 4 D

HEK(LHON)	+MTS8k_ND4	+MTS8n_ND4	+MTS10_ND4	+MTS4_ND4	+MTS DNAjc30_ND4	+MTS 8k_ND4mut
1070,8	1430,5	1742,8	1200,7	1696,8	1841,3	2209,6
1021,6	1546,1	1722,8	1182,7	1926,9	1725,4	2390,7
1169,9	1578,1	1866	1243,2	1731,3	1996,8	2238,3
1147,2	1484	1590,4	1165,6	1605,4	1732,2	2241,2

Table S13 - Data for Figure 4 F

HEK(LHON)	+MTS8k_ND4	+MTS8n_ND4	+MTS10_ND4	+MTS4_ND4	+MTS DNAjc30_ND4	+MTS 8k_ND4mut
11401,6	19875,6	22352,2	21732,6	19246,4	18538,6	16816,1
12086,5	20003,4	21222,7	21795,5	19068,8	16739,5	15123,5
10343,3	19331,2	21279,4	19680,3	18426,8	16661,3	15839,6
12365,7	19279,6	22427,5	19799,1	18390,6	16675,7	15412,1

4 MitoFates comparison of MTS under study

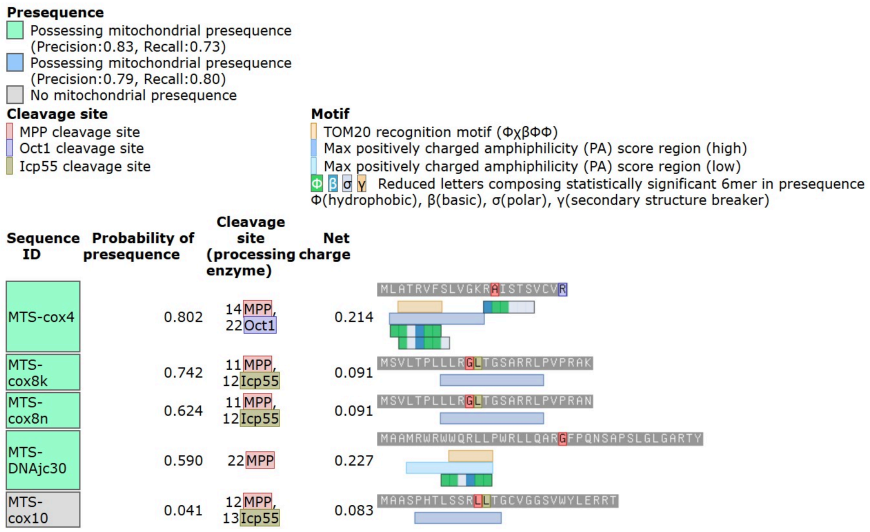

Supplement: Supplementary file 1 [file DataSheet1.pdf]
